# Supplementary material for: Recombinant Protein Spectral Library (rPSL) DIA-MS method improves identification and quantification of low-abundance cancer-associated and kynurenine pathway proteins
Source: Commun Chem. 2025 May 10;8:141. doi: 10.1038/s42004-025-01531-0 (PMC12065878; doi:10.1038/s42004-025-01531-0)

Supplementary Information

Recombinant Protein Spectral Library (rPSL) DIA-MS Method Improves Identification and Quantification of Low-Abundance Cancer-Associated and Kynurenine Pathway Proteins

Shivani Krishnamurthy[1], Bavani Gunasegaran[1], Moumita Paul-Heng[2], Abidali Mohamedali[1,3], William P.Klare[4], C.N.Ignatius Pang[4], Laurence Gluch[1,5], Joo-Shik Shin[6,7], Charles Chan[8,9], Mark S Baker[1], Seong Beom Ahn[1]* and Benjamin Heng[1]*

[1] Macquarie Medical School, Faculty of Medicine, Health and Human Sciences, Macquarie University, Sydney, Australia
[2] Transplantation Immunobiology Research Group, Charles Perkins Centre, The University of Sydney, Sydney, Australia
[3] Faculty of Science and Engineering, School of Natural Sciences, Macquarie University, Sydney, Australia
[4] Australian Proteome Analysis Facility, Macquarie University, Sydney, Australia
[5] The Strathfield Breast and Thyroid Centre, Strathfield, Sydney, Australia
[6] Department of Tissue Pathology and Diagnostic Oncology, Royal Prince Alfred Hospital, Camperdown, Sydney, Australia.
[7] Central Clinical School, Faculty of Medicine and Health, The University of Sydney, Sydney, Australia
[8] Department of Anatomical Pathology, NSW Health Pathology, Concord Hospital, Sydney, NSW, Australia.
[9] Concord Institute of Academic Surgery, Concord Clinical School, Faculty of Medicine and Health, Concord Hospital, The University of Sydney, Sydney, Australia

Corresponding Authors*
Dr. Benjamin Heng, PhD, Faculty of Medicine, Health and Human Sciences, Macquarie University
Dr. Seong Beom Ahn, PhD, Faculty of Medicine, Health and Human Sciences, Macquarie University

Email : Benjamin.heng@mq.edu.au, Charlie.ahn@mq.edu.au

Table of Content

File Name	Description	Page Number
Supplementary Table 1	List of 42 human recombinant proteins included in this study, along with their molecular weight information.	**S1**
Supplementary Table 2	A table representing the total number of peptides and proteins quantified using DIANN across the four workflows - biological-rPSL, rPSL, biological-library and library-free DIA-MS analysis for the tissue and cell lysate experiments.	**S2**
Supplementary Figure 1	MS2 Extracted Ion Chromatograms (XICs) and MS2 spectra at Apex for selected peptides using Spectronaut across four DIA-MS workflows. The XICs illustrate the retention time, peak intensities and chromatographic profile of different fragments for a selected peptide, as defined in the spectral libraries. The full recorded MS2 spectrum corresponds to the apex of the peptide's chromatographic peak, with the detected fragment ions highlighted.	**S3 - S5**
Supplementary Figure 2	Analyses of protein intensities across library-free DIA, biological-library DIA, biological-rPSL DIA, and rPSL DIA-MS methods for proteins (S100A9, ITGAV and PFN1) quantified in both noncancerous and tumour tissues, and CEACAM5, TP53, MMP2, MUC1 and TIMP1 proteins quantified exclusively quantified in tumour tissues only.	**S6**
Supplementary Figure 3	Bar charts illustrating statistically significant proteins identified between control and IFN-γ treated cells, measured in triplicate.	**S7**

Supplementary Table 1

Protein ID	Gene Name	Protein Description	Molecular weight (kDa)	Detected using MS (Yes or No)*
Q8N5Z0	**AADAT**	Kynurenine/alpha-aminoadipate aminotransferase, mitochondrial	48	Yes
P35070	**BTC**	Probatecellulin	19.75	Yes
P02747	**C1QC**	Complement C1q subcomponent subunit C	25.77	Yes
Q99626	**CDX2**	Homeobox protein CDX-2	34	Yes
P06731	**CEACAM5**	Carcinoembryonic antigen-related cell adhesion molecule 5	76.8	Yes
Q9Y646	**CPQ**	Carboxypeptidase Q	51.89	Yes
P02778	**CXCL10**	C-X-C motif chemokine 10	10.88	Yes
P48061	**CXCL12**	Stromal cell-derived factor 1	10.67	Yes
P10145	**CXCL8**	Interleukin-8	11.1	Yes
P04798	**CYP1A1**	Cytochrome P450 1A1	58	Yes
Q16678	**CYP1B1**	Cytochrome P450 1B1	36	Yes
P01133	**EGF**	Pro-epidermal growth factor	6.22	Yes
P14902	**IDO1**	Indoleamine 2,3-dioxygenase 1	46	Yes
Q6ZQW0	**IDO2**	Indoleamine 2,3-dioxygenase 2	46	No
P01584	**IL1B**	Interleukin-1 beta	30.75	Yes
P05231	**IL6**	Interleukin-6	23.72	No
P06756	**ITGAV**	Integrin Alpha V	116.04	Yes
P05556	**ITGB1**	Integrin Beta-1	88.42	Yes
P18564	**ITGB6**	Integrin Beta-6	85.94	Yes
P07288	**KLK3**	Prostate-specific antigen	28.74	No
O15229	**KMO**	Kynurenine 3-monooxygenase	51	Yes
P35900	**KRT20**	Keratin, type I cytoskeletal 20	48.48	Yes
Q16719	**KYNU**	Kynureninase	54	Yes
Q16674	**MIA**	Melanoma-derived growth regulatory protein	14.51	Yes
P08253	**MMP2**	72 kDa type IV collagenase	73.88	Yes
P08254	**MMP3**	Stromelysin-1	53.98	Yes
P14780	**MMP9**	Matrix metalloproteinase 9	93	Yes
P15941	**MUC1**	Mucin-1	122.1	Yes
P01127	**PDGFB**	Platelet-derived growth factor subunit B	27.28	Yes
P07737	**PFN1**	Profilin-1	15.05	Yes
Q03405	**PLAUR**	Urokinase plasminogen activator surface receptor	36.98	Yes
P60484	**PTEN**	Phosphatidylinositol 3,4,5-trisphosphate 3-phosphatase and dual-specificity protein phosphatase	47.17	Yes
Q15274	**QPRT**	Nicotinate-nucleotide pyrophosphorylase [carboxylating]	30.6	Yes
P05109	**S100A8**	Protein S100-A8	10.84	Yes
P06702	**S100A9**	Protein S100-A9	13.24	Yes
P48775	**TDO2**	Tryptophan 2,3-dioxygenase	45	Yes
P01135	**TGFA**	Transforming growth factor alpha	17.01	Yes
P01033	**TIMP1**	Metalloproteinase inhibitor 1	23.17	Yes
P16035	**TIMP2**	Metalloproteinase inhibitor 2	24.4	Yes
P01375	**TNF**	Tumor necrosis factor	25.64	Yes
P19438	**TNFRSF1A**	Tumor necrosis factor receptor superfamily member 1A	50.5	Yes
P04637	**TP53**	Cellular tumor antigen p53	43.65	Yes

**based on data available on the National Cancer Institute Clinical Proteomic Tumor Analysis Consortium (CPTAC) https://pdc.cancer.gov*

Supplementary Table 2

Tissue experiments	Analysis 1	Analysis 2	Analysis 3	Analysis 4
	rPSL-DIA	Tissue Biological-rPSL DIA	Tissue biological-library DIA	Library free DIA
Number of proteins quantified	156	7036	7025	6445
Number of peptides quantified	837	45048	44869	36122

Cell lysate experiments	Analysis 1	Analysis 2	Analysis 3	Analysis 4
	rPSL-DIA	Cell Biological-rPSL DIA	Cell biological-library DIA	Library free DIA
Number of proteins quantified	198	8402	8370	7422
Number of peptides quantified	1021	67185	67193	51804

Supplementary Figure 1

(A) Peptide Sequence - **NIETIINTFHQYSVK**

Sample-specific biological-library based DIA-MS

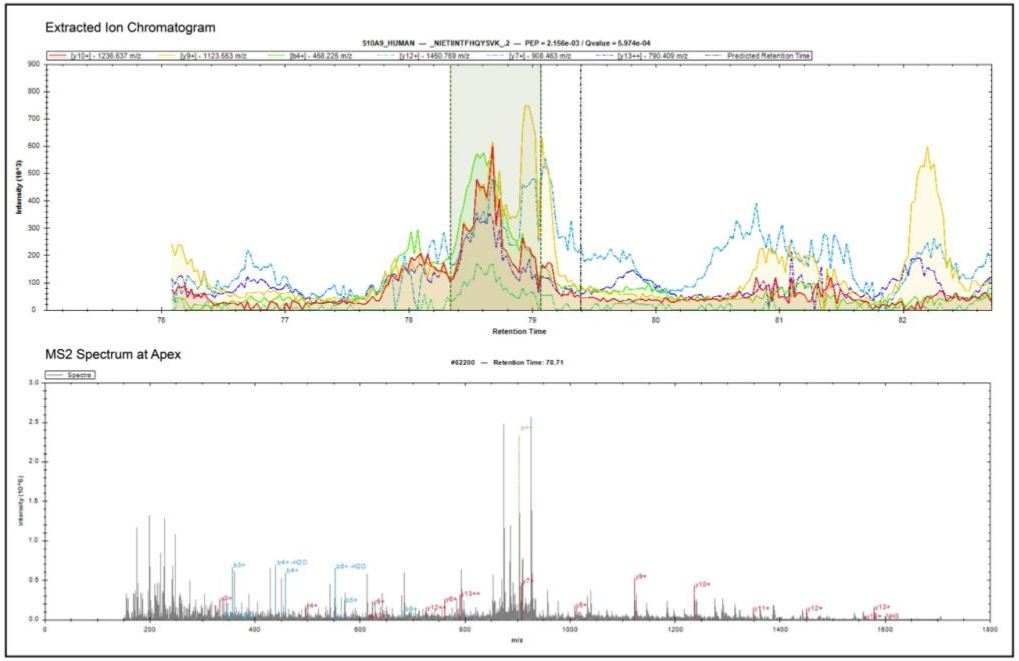

Recombinant protein spectral library based DIA-MS

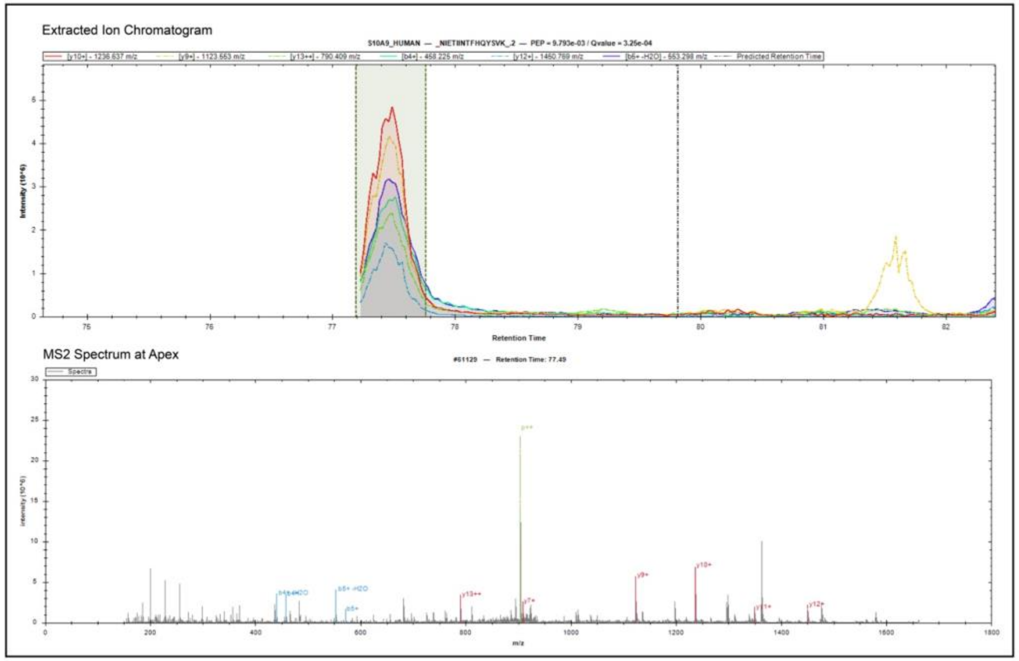

Library-free DIA-MS

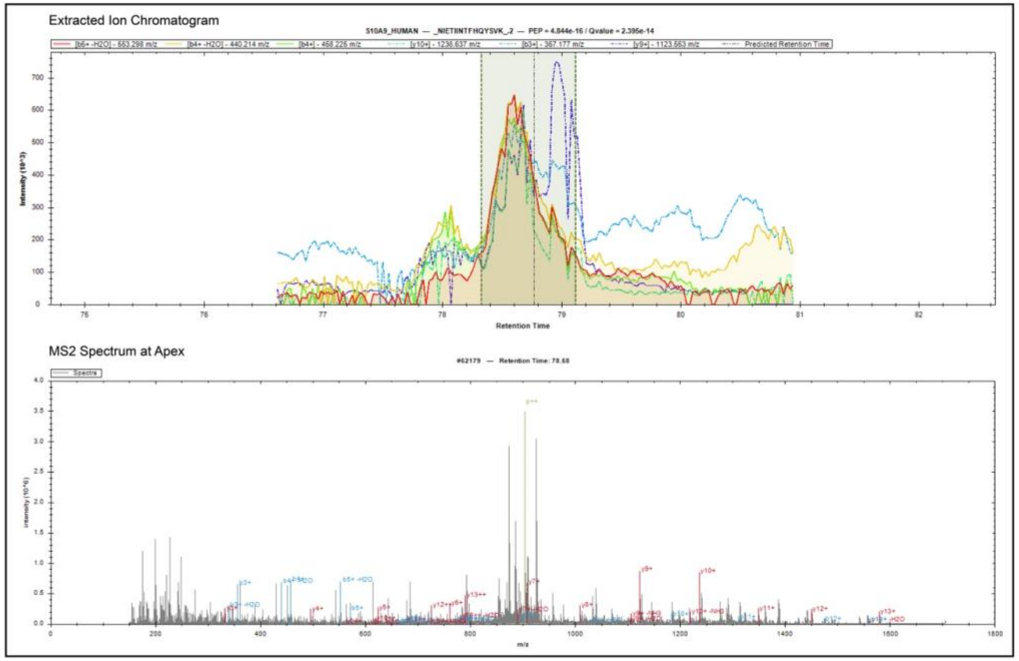

Sample-specific biological-rPSL based DIA-MS

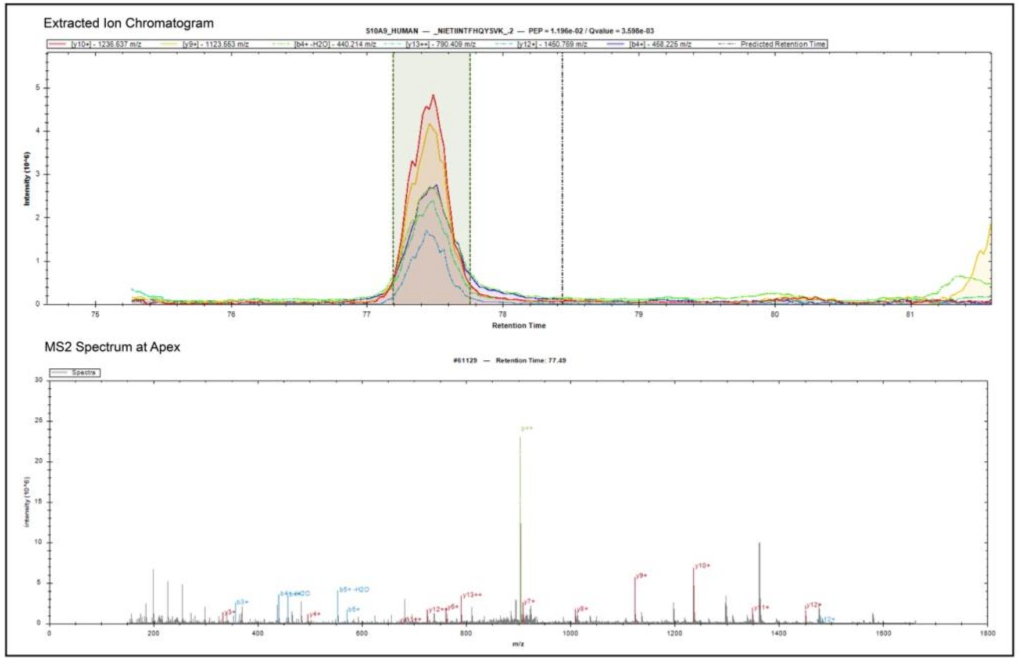

(B) Peptide Sequence - **TDKTLVLLMGK**

Sample-specific biological-library based DIA-MS

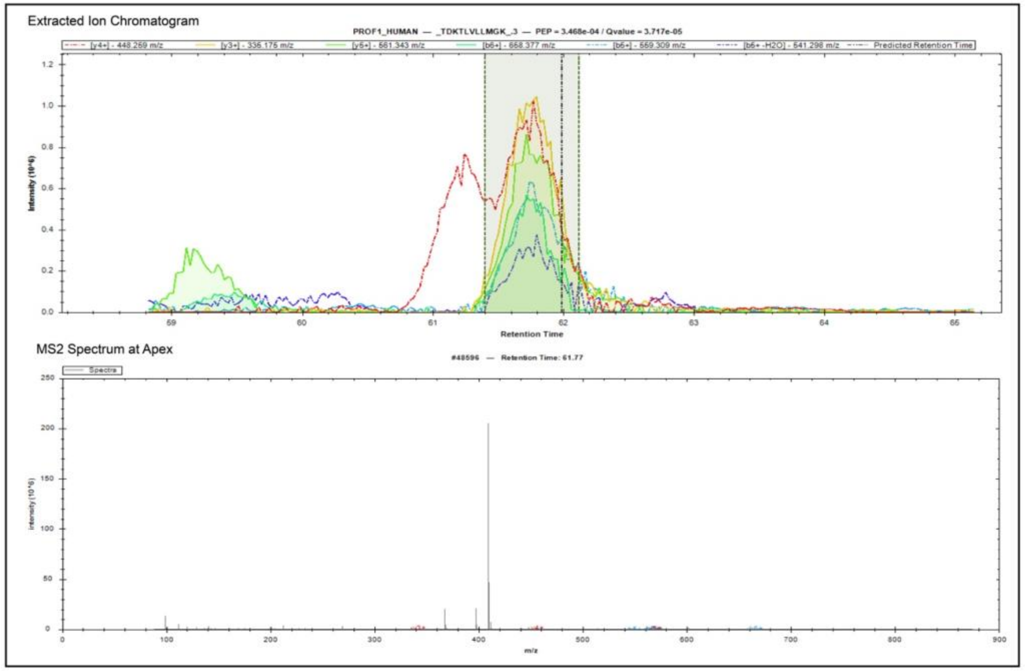

Recombinant protein spectral library based DIA-MS

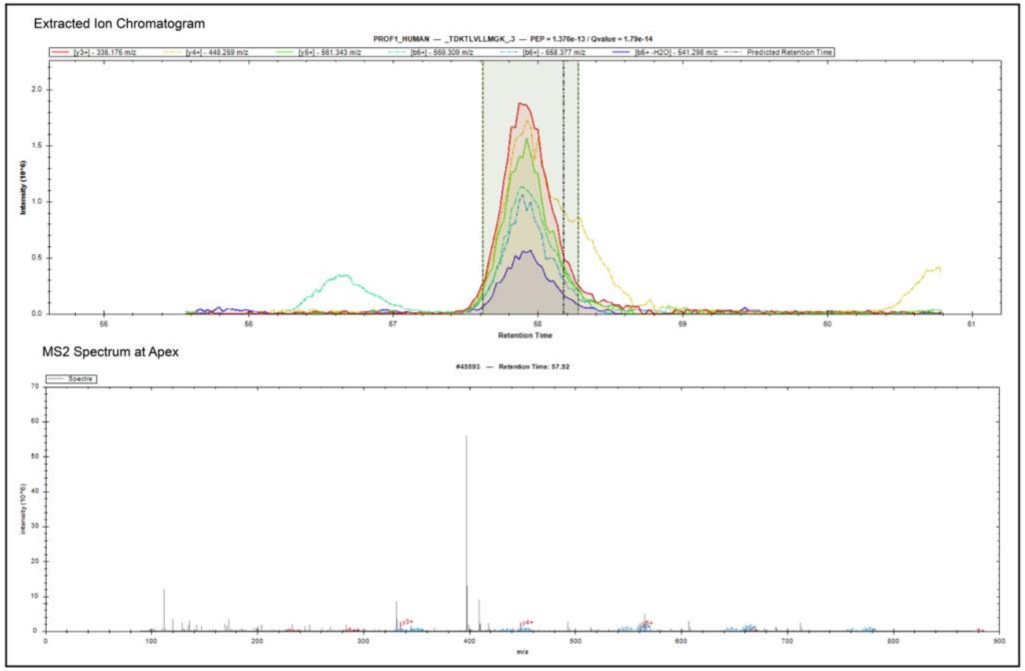

Library-free DIA-MS

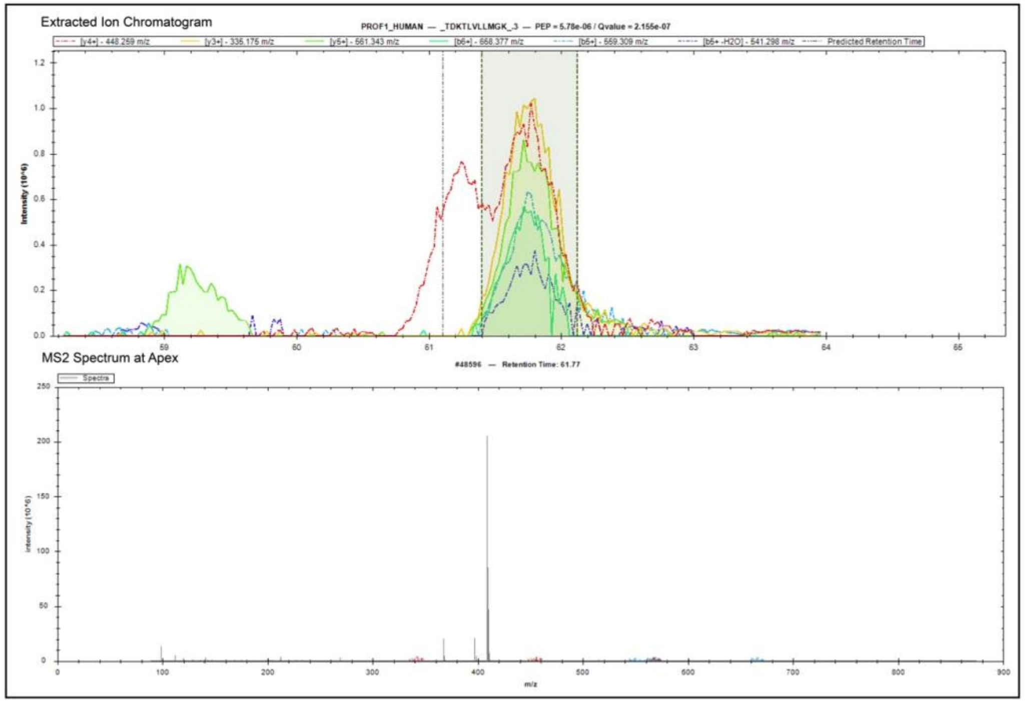

Sample-specific biological-rPSL based DIA-MS

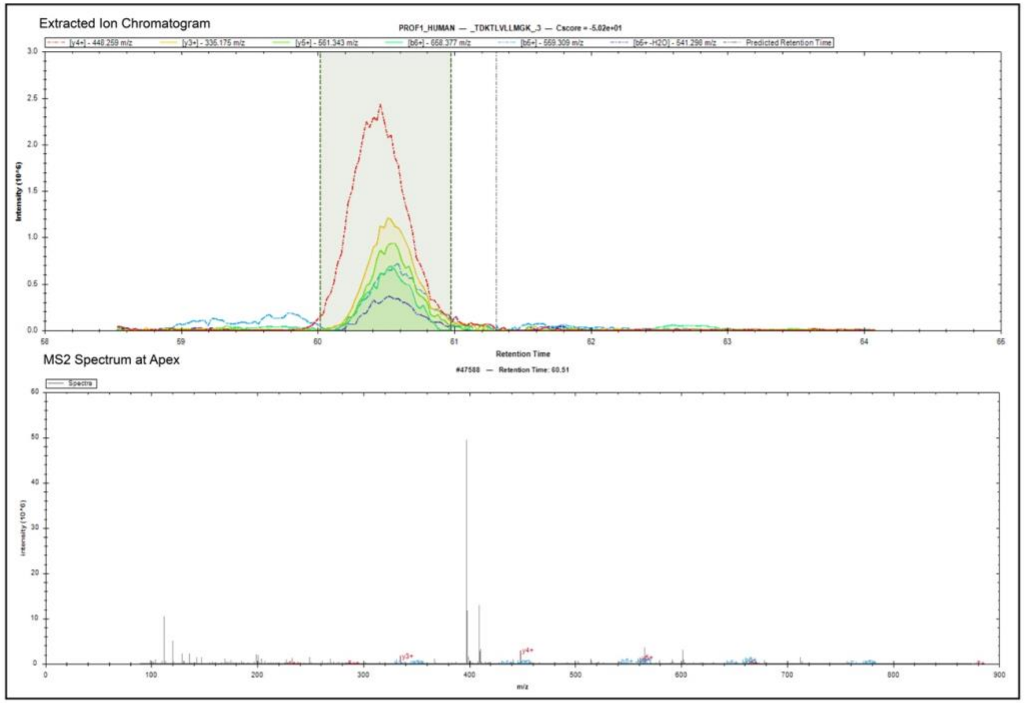

(C) Peptide Sequence - **KKLETECPQYIR**

Sample-specific biological-library based DIA-MS

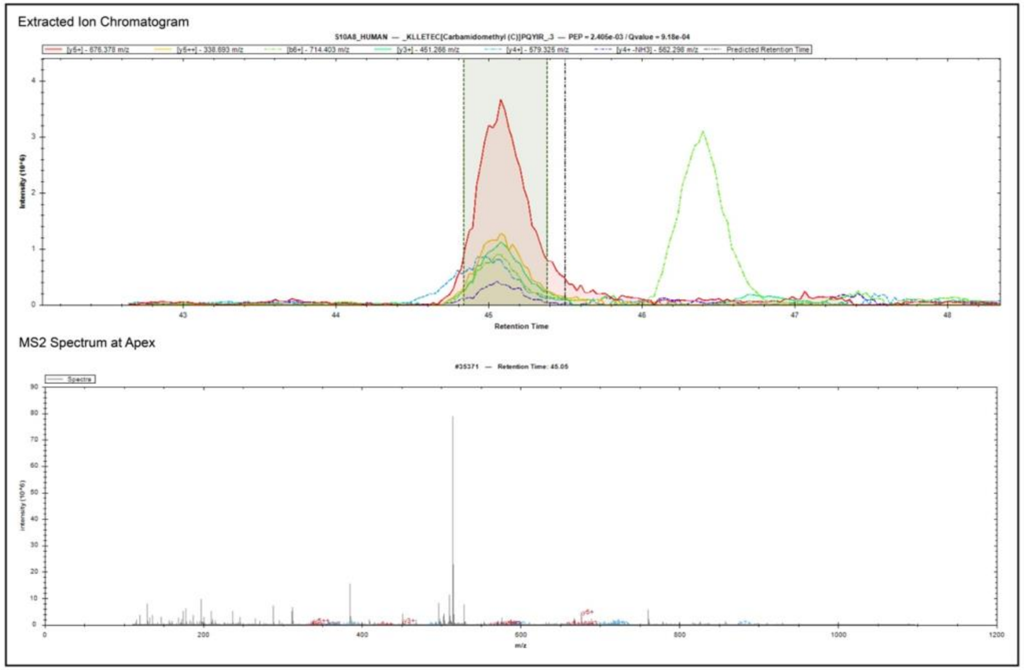

Recombinant protein spectral library based DIA-MS

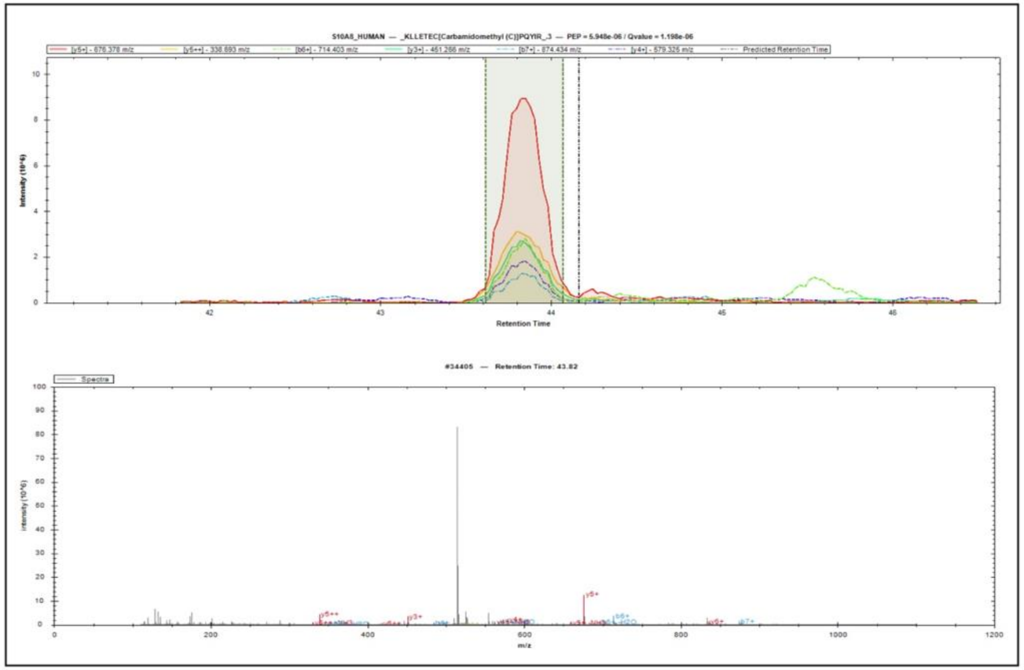

Library-free DIA-MS

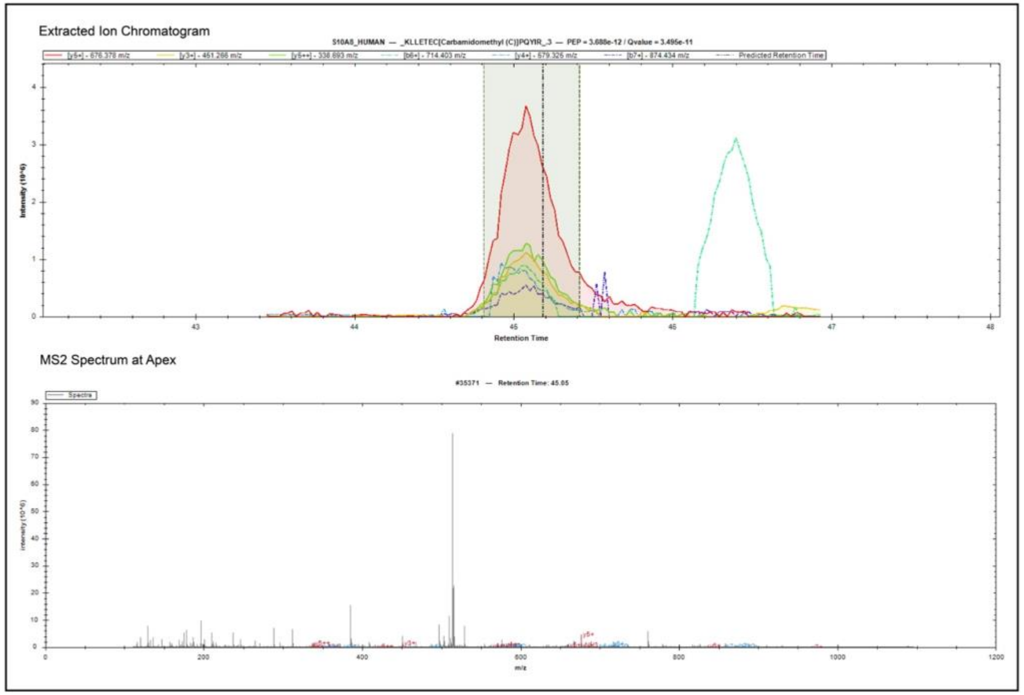

Sample-specific biological-rPSL based DIA-MS

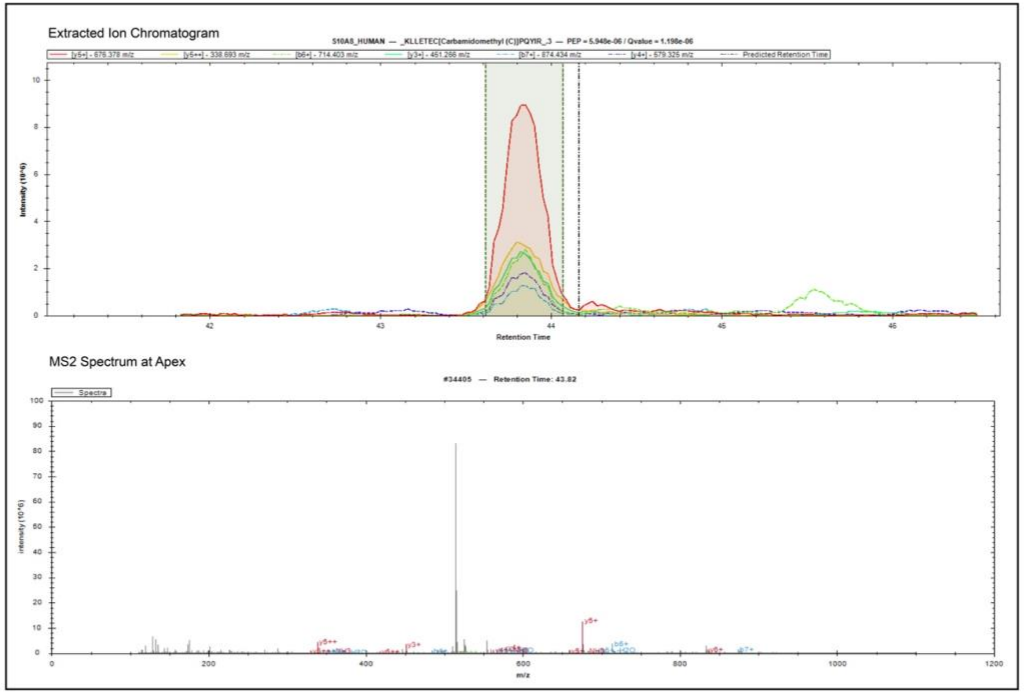

Supplementary Figure 2

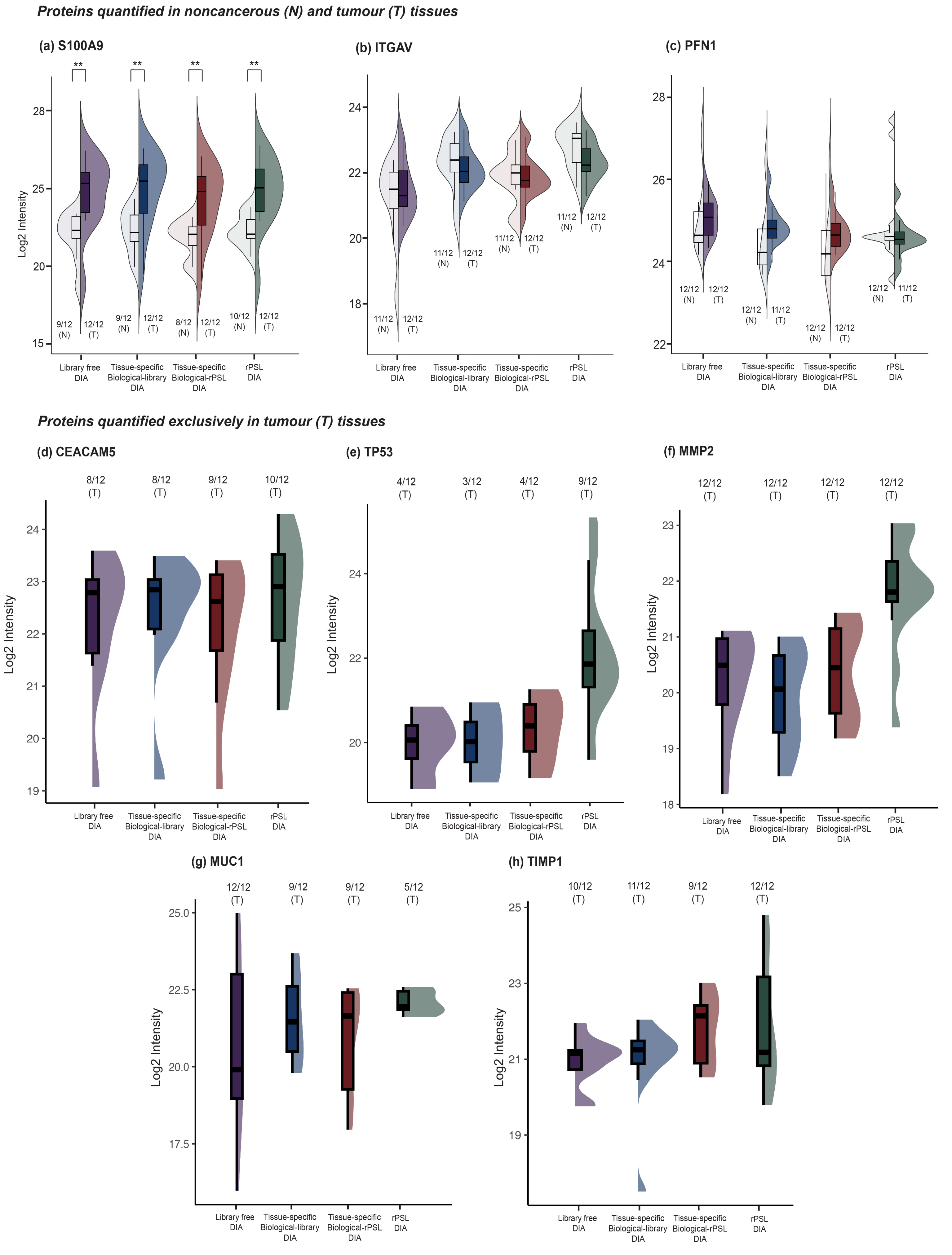

Supplementary Figure 3

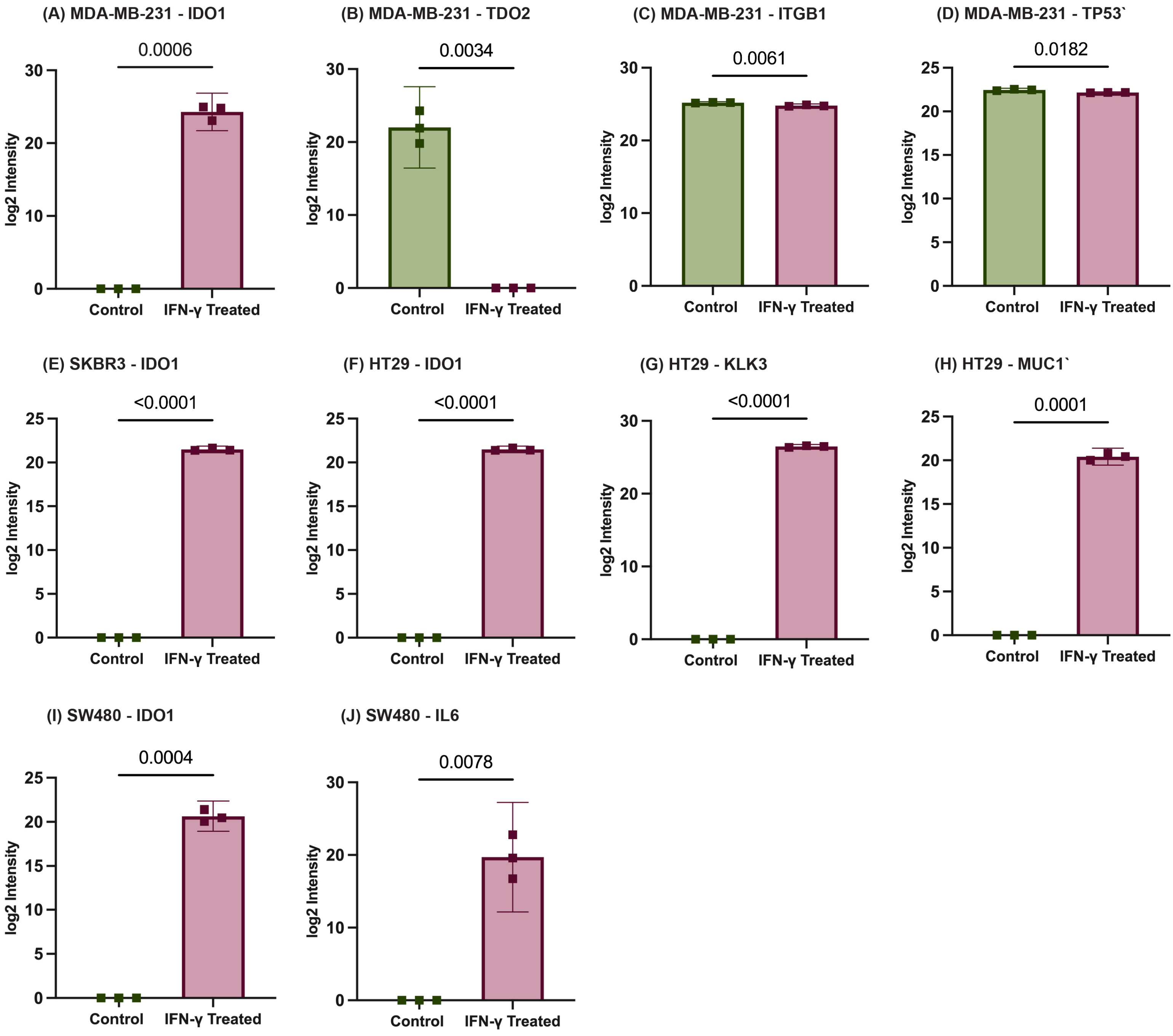

Supplement: Supplementary file 2 — Supplementary Information [file 42004_2025_1531_MOESM2_ESM.pdf]
