# Supplementary material for: Recombinant Protein Spectral Library (rPSL) DIA-MS method improves identification and quantification of low-abundance cancer-associated and kynurenine pathway proteins
Source: Commun Chem. 2025 May 10;8:141. doi: 10.1038/s42004-025-01531-0 (PMC12065878; doi:10.1038/s42004-025-01531-0)
Supplement: Supplementary file 3 — Description of Additional Supplementary Files [file 42004_2025_1531_MOESM3_ESM.pdf]

Description of Additional Supplementary files

Supplementary Data 1
Description : Three spectral libraries (Tissue biological-library, rPSL and Tissue biological-rPSL) generated using the Fragpipe platform, utilized for DIA-MS analysis in tissue experiments, including a list of all identified proteins (filtered at ≥ 99% confidence probability) and the corresponding number of peptides per protein

Supplementary Data 2
Description : Three spectral libraries (Cell biological-library, rPSL and Cell biological-rPSL) generated using the Fragpipe platform, utilized for DIA-MS analysis in cell lysate experiments, including a list of all identified proteins (filtered at ≥ 99% confidence probability) and the corresponding number of peptides per protein.

Supplementary Data 3
Description : A list of unique peptides identified per protein for the 42 proteins included in the study across the three spectral libraries (Tissue biological-library, rPSL and Tissue biological-rPSL) for the tissue experiments.

Supplementary Data 4
Description : A list of unique peptides identified per protein for the 42 proteins included in the study across the three spectral libraries (Cell biological-library, rPSL and Cell biological-rPSL) for the cell lysate experiments.

Supplementary Data 5
Description : A list of unique peptides detected or quantified per protein for the 42 proteins analysed across the four workflows - biological-rPSL, rPSL, biological-library, and library-free DIA-MS analysis for the tissue experiments, using DIANN

Supplementary Data 6
Description : A list of unique peptides detected or quantified per protein for the 42 proteins across the four workflows - biological-rPSL, rPSL, biological-library, and library-free DIA-MS analysis for the cell lysate experiments, using DIANN

Supplementary Data 7
Description : A list of peptides exclusively detected using biological-rPSL DIA-MS approach, as well as peptides commonly identified across standard DIA-MS data analysis methods (biological-library-DIA and library-free DIA) and biological-rPSL-DIA, using DIANN

Supplementary Data 8
Description : Spectronaut Data Analysis. This file contains a table summarizing the total number of peptides and proteins identified in three spectral libraries generated using Pulsar Search on Spectronaut, as well as the total number of proteins and peptides quantified using Spectronaut across four DIA-MS data extraction workflows - biological-rPSL, rPSL, biological-library and library-free (DirectDIA) DIA-MS analysis for both tissue and cell lysate experiments. Additionally, it includes tables presenting the number of peptides detected per protein for the 42 proteins analysed.

Supplementary Data 9
Description : Log2 protein abundance calculated in matched breast and colorectal cancer tissue samples across the four workflows—biological-rPSL, rPSL, biological-library, and library-free DIA-MS analysis for the tissue experiments.

Supplementary Data 10
Description : Log2 protein abundance calculated in five cancer cell lines—control, vehicle control, and interferon-gamma-treated cell lysates—across the four workflows: biological-rPSL, rPSL, biological-library, and library-free DIA-MS analysis.

Supplementary Data 11
Description : Dataset including the fold change ratios for each tumour sample for the 42 proteins, comparing tumour tissues to their matched adjacent noncancerous tissues, along with their log2 fold change ratios.

Supplementary Data 12
Description : Dataset containing the protein intensities for the 42 proteins calculated in the cell lines, including imputed data and the results from a Welch's t-test comparing treated to untreated cells.
