# Supplementary material for: Recombinant Protein Spectral Library (rPSL) DIA-MS method improves identification and quantification of low-abundance cancer-associated and kynurenine pathway proteins
Source: Commun Chem. 2025 May 10;8:141. doi: 10.1038/s42004-025-01531-0 (PMC12065878; doi:10.1038/s42004-025-01531-0)
Supplement: Supplementary file 16 — Reporting Summary [file 42004_2025_1531_MOESM16_ESM.pdf]

nature portfolio

Corresponding author(s): Shivani Krishnamurthy

Last updated by author(s): Apr 17, 2025

Reporting Summary

Nature Portfolio wishes to improve the reproducibility of the work that we publish. This form provides structure for consistency and transparency in reporting. For further information on Nature Portfolio policies, see our Editorial Policies and the Editorial Policy Checklist.

Statistics

For all statistical analyses, confirm that the following items are present in the figure legend, table legend, main text, or Methods section.

n/a	Confirmed	
☐	☒	The exact sample size (*n*) for each experimental group/condition, given as a discrete number and unit of measurement
☐	☒	A statement on whether measurements were taken from distinct samples or whether the same sample was measured repeatedly
☐	☒	The statistical test(s) used AND whether they are one- or two-sided<br>*Only common tests should be described solely by name; describe more complex techniques in the Methods section.*
☒	☐	A description of all covariates tested
☒	☐	A description of any assumptions or corrections, such as tests of normality and adjustment for multiple comparisons
☐	☒	A full description of the statistical parameters including central tendency (e.g. means) or other basic estimates (e.g. regression coefficient) AND variation (e.g. standard deviation) or associated estimates of uncertainty (e.g. confidence intervals)
☐	☒	For null hypothesis testing, the test statistic (e.g. *F*, *t*, *r*) with confidence intervals, effect sizes, degrees of freedom and *P* value noted<br>*Give P values as exact values whenever suitable.*
☒	☐	For Bayesian analysis, information on the choice of priors and Markov chain Monte Carlo settings
☒	☐	For hierarchical and complex designs, identification of the appropriate level for tests and full reporting of outcomes
☒	☐	Estimates of effect sizes (e.g. Cohen's *d*, Pearson's *r*), indicating how they were calculated

Our web collection on statistics for biologists contains articles on many of the points above.

Software and code

Policy information about availability of computer code

Data collection	The samples were acquired using two modes. For spectral library generation, the samples were acquired on Data Dependent Acquisition (DDA) mode. Each individual sample was acquired on a Data Independent Acquisition (DIA) mode. A Vanquish UHPLC system coupled to an Orbitrap Exploris 480 mass spectrometer (ThermoFisher Scientific, MA, USA) was used in this study
Data analysis	Raw DIA-MS datasets were processed using DIA-NN (version 1.8.1) and Spectronaut (version 19.5). Fragpipe platform (version 22.0) was used for spectral library generation for DIANN library-based analysis.

For manuscripts utilizing custom algorithms or software that are central to the research but not yet described in published literature, software must be made available to editors and reviewers. We strongly encourage code deposition in a community repository (e.g. GitHub). See the Nature Portfolio guidelines for submitting code & software for further information.

Data

Policy information about availability of data

All manuscripts must include a data availability statement. This statement should provide the following information, where applicable:

- Accession codes, unique identifiers, or web links for publicly available datasets
- A description of any restrictions on data availability
- For clinical datasets or third party data, please ensure that the statement adheres to our policy

The mass spectrometry proteomics data have been deposited to the ProteomeXchange Consurtium via the PRIDE partner repository with the dataset identifier PXD061696

Research involving human participants, their data, or biological material

Policy information about studies with human participants or human data. See also policy information about sex, gender (identity/presentation), and sexual orientation and race, ethnicity and racism.

Reporting on sex and gender	Sex and gender information of the patients were not included in the study design. This study focuses on demonstrating the feasibility of a proteomics method developed to quantify lower-abundance proteins across various sample matrices. No biological interpretations or findings were derived from this study
Reporting on race, ethnicity, or other socially relevant groupings	n/a
Population characteristics	Breast Cancer - All breast cancer patients were female with a mean age of 58 years (range from 40 to 82 years). All had grade 3 tumour, comprising of 2 triple negative, 1 luminal B and 2 HER2-positive molecular subtypes.<br>Colorectal Cancer - 57% of the colorectal cancer patients were female, and the remainder were male. The mean age was 55 years (range from 30 to 80 years). The cohort included 4 patients with early-stage and 3 with late-stage disease.
Recruitment	The Colorectal Cancer samples were purchased from Victorian Cancer Biobank, Melbourne.<br><br>Breast Cancer samples<br>Recruitment protocol adherence to Australian National Statement<br>i. No nurse from the participating hospital is involved in this research project and has no financial or contractual relationship with any researchers involved in this project.<br>ii. Biological samples will only collected from consent donor who has made a decision to participate and is deemed safe and within the legal terms to have the operation/collection by a registered medical practitioner or phlebotomist.<br>iii. Only the clinic nurse is involved in the recruitment.<br>vi.The clinic nurse will emphasize that their decision will not have any impact on their well-being, care (before and after operation, if any required), and the operation itself. This information is also mentioned in the patient information/consent form.<br>v. Potential donor is given an opportunity to consider/review this information at least a week to consider/understand the study before contacting the clinician nurse. They have a choice to not proceed with the donation in their followup appointment or prior to operation (if any required) or commencement of their participation.<br>vi. Only patients who can communicate and understand English are recruited for this study.<br>vii. The biospecimens will be used for research purposes only in accordance with the consent obtained from patients, as mentioned in the patient consent/information form
Ethics oversight	This study was approved by the Macquarie University Human Research Ethics Committee (Medical Sciences).

Note that full information on the approval of the study protocol must also be provided in the manuscript.

Field-specific reporting

Please select the one below that is the best fit for your research. If you are not sure, read the appropriate sections before making your selection.

☒ Life sciences ☐ Behavioural & social sciences ☐ Ecological, evolutionary & environmental sciences

For a reference copy of the document with all sections, see nature.com/documents/nr-reporting-summary-flat.pdf

Life sciences study design

All studies must disclose on these points even when the disclosure is negative.

Sample size	No sample size calculation was performed. The sample size of n=24 was used to demonstrate the methodology developed.
Data exclusions	No data was excluded

Replication	Biological replicates were used to demonstrate the sensitivity and robustness of the method developed to quantify lower-abundance cancer-associated and kynurenine pathway proteins in complex biological matrices
Randomization	Not relevant to our study. This study focuses on demonstrating the feasibility of the proteomics method developed. No biological interpretations or findings were derived from this study
Blinding	Blinding was not relevant to this study, which focuses on proteomics method development. No biological interpretation was derived from the data

Reporting for specific materials, systems and methods

We require information from authors about some types of materials, experimental systems and methods used in many studies. Here, indicate whether each material, system or method listed is relevant to your study. If you are not sure if a list item applies to your research, read the appropriate section before selecting a response.

Materials & experimental systems

n/a	Involved in the study	
☒	☐	Antibodies
☐	☒	Eukaryotic cell lines
☒	☐	Palaeontology and archaeology
☒	☐	Animals and other organisms
☒	☐	Clinical data
☒	☐	Dual use research of concern
☒	☐	Plants

Methods

n/a	Involved in the study	
☒	☐	ChIP-seq
☒	☐	Flow cytometry
☒	☐	MRI-based neuroimaging

Eukaryotic cell lines

Policy information about cell lines and Sex and Gender in Research

Cell line source(s)	All cell lines used in this study were purchased from the American Type Culture Collection (ATCC) and are of human origin
Authentication	None of the cell lines used were authenticated
Mycoplasma contamination	Cell lines were not tested for mycoplasma contamination
Commonly misidentified lines (See ICLAC register)	None

Plants

Seed stocks	n/a
Novel plant genotypes	n/a
Authentication	n/a
